# Supplementary material for: Using whole-genome sequence data to examine the epidemiology of antimicrobial resistance in Escherichia coli from wild meso-mammals and environmental sources on swine farms, conservation areas, and the Grand River watershed in southern Ontario, Canada
Source: PLoS One. 2022 Apr 8;17(4):e0266829. doi: 10.1371/journal.pone.0266829 (PMC8993012; doi:10.1371/journal.pone.0266829)
Supplement: S1 Table — (DOCX) [file pone.0266829.s002.docx]

**Supplementary Table S1: Multi-locus sequence types identified using whole-genome sequence data of phenotypically resistant *Escherichia coli* isolates obtained from wildlife, swine manure pits, and environmental sources in southern Ontario, Canada, 2011−2013 (n=200)**

| **Sequence type** | **Count (%)** |
| --- | --- |
| 58 | 15 (7.5%) |
| 10 | 14 (7.0%) |
| 101 | 12 (6.0%) |
| 69 | 8 (4.0%) |
| 155 | 7 (3.5%) |
| 542 | 5 (2.5%) |
| 345 | 5 (2.5%) |
| 648 | 4 (2.0%) |
| 1633 | 4 (2.0%) |
| 898 | 3 (1.5%) |
| 3714 | 3 (1.5%) |
| 1727 | 3 (1.5%) |
| 34 | 3 (1.5%) |
| 43 | 3 (1.5%) |
| 12 | 3 (1.5%) |
| 1844 | 3 (1.5%) |
| 48 | 3 (1.5%) |

*Sequence types identified in fewer than 3 isolates were: 131, 295, 963, 1304, 847, 162, 354, 1721, 127, 515, 7324, 196, 641, 349, 93, 4085, 388, 547, 683, 5614, 646, 362, 602, 372, 906, 2307, 4574, 68, 2526, 2171, 6796, 6706, 2562, 2035, 457, 297, 3076, 6975, 1406, 9962, 164, 3856, 2077, 38, 4398, 88, 8097, 871, 227, 2354, 2178, 23, 9982, 3531, 1079, 117, 398, 1152, 1324, 106, 218, 154, 1585, 962, 4429, 1086, 212, 1670, 357, 1112, 716, 5259, 95, 973, 6777, 206.
