# Supplementary material for: Using whole-genome sequence data to examine the epidemiology of antimicrobial resistance in Escherichia coli from wild meso-mammals and environmental sources on swine farms, conservation areas, and the Grand River watershed in southern Ontario, Canada
Source: PLoS One. 2022 Apr 8;17(4):e0266829. doi: 10.1371/journal.pone.0266829 (PMC8993012; doi:10.1371/journal.pone.0266829)
Supplement: S2 Table — (DOCX) [file pone.0266829.s003.docx]

**Supplementary Table S2: Serotypes identified using whole-genome sequence data of phenotypically resistant *Escherichia coli* isolates obtained from wildlife, swine manure pits, and environmental sources in southern Ontario, Canada, 2011−2013 (n=200)**

| **Serotype** | **Count** |
| --- | --- |
| O82:H8 | 7 |
| O24:H4 | 6 |
| O153:H42 | 4 |
| O46/O8/O134:H21 | 3 |
| O42:O28:H21 | 3 |
| O8:H49 | 3 |
| O83:H14 | 3 |
| O4:H5 | 3 |
| O6:H10 | 3 |
| O109:H10 | 3 |
| O77/O17/O44/O106:H18 | 3 |
| O2:H18 | 2 |
| O166:H15 | 2 |
| O108:H2 | 2 |
| O91:H7 | 2 |
| O1:H34 | 2 |

*****Additional serotypes not listed here were identified in fewer than two isolates. The O antigen could not be predicted for 38 isolates. One isolate could not be serotyped.
