# Supplementary material for: Using whole-genome sequence data to examine the epidemiology of antimicrobial resistance in Escherichia coli from wild meso-mammals and environmental sources on swine farms, conservation areas, and the Grand River watershed in southern Ontario, Canada
Source: PLoS One. 2022 Apr 8;17(4):e0266829. doi: 10.1371/journal.pone.0266829 (PMC8993012; doi:10.1371/journal.pone.0266829)
Supplement: S3 Table — (DOCX) [file pone.0266829.s004.docx]

**Supplementary Table S3: Distribution of plasmid incompatibility types identified using whole-genome sequence data by source type for phenotypically resistant *Escherichia coli* isolates from wildlife, swine manure pits, and environmental sources in southern Ontario, Canada, 2011−2013 (n=200)**

| **Inc-type** | **Source Type** | | | | **Total^c^ (%)** |
| --- | --- | --- | --- | --- | --- |
|  | **Wildlife^a^ (n=72)** | **Swine Manure Pit (n=31)** | **Water (n=20)** | **Other Environmental^b^ (n=76)** |  |
| IncFIB(AP001918) | 30 | 7 | 9 | 33 | 79 (39.5%) |
| IncI1(1-alpha) | 14 | 4 | 1 | 13 | 32 (16.0%) |
| IncFII | 8 | 7 | 4 | 10 | 29 (14.5%) |
| IncFIA | 7 | 4 | 2 | 6 | 19 (9.5%) |
| p0111 | 7 | 7 | 2 | 3 | 19 (9.5%) |
| IncY | 3 | 5 | 1 | 8 | 17 (8.5%) |
| IncQ1 | 3 | 1 | 4 | 6 | 14 (7.0%) |
| IncX1-1 | 6 | 2 | 1 | 3 | 12 (6.0%) |
| Col156 | 5 | 0 | 4 | 1 | 10 (5.0%) |

^a^ Includes fecal isolates from raccoons (n=51), skunks (n=4), opossums (n=2), and paw swab samples from raccoons (n=15), and one skunk.

^b^ Includes soil (n=73) and dumpster isolates (n=3).

^c^ Plasmid Inc types identified in fewer than 10 isolates included: IncR (n=9), IncFIA(HI1) (n=6), IncFIC(FII) (n=5), IncFIB(K) (n=4), IncFII(29), (n=4), IncHI2A (n=3), IncHI2 (n=3), IncC (n=3), IncB/O/K/Z (n=2), IncFII(pHN7A8) (n=2), ColBS512 (n=2), ColE10 (n=2), ColpVC (n=2), IncFIB(pB171) (n=1), ColIMGS31 (n=1), IncFII(pRSB107) (n=1), IncHI1A(CIT) (n=1), IncHI1B(CIT) (n=1), IncX1-4 (n=1).
