# Supplementary material for: Using whole-genome sequence data to examine the epidemiology of antimicrobial resistance in Escherichia coli from wild meso-mammals and environmental sources on swine farms, conservation areas, and the Grand River watershed in southern Ontario, Canada
Source: PLoS One. 2022 Apr 8;17(4):e0266829. doi: 10.1371/journal.pone.0266829 (PMC8993012; doi:10.1371/journal.pone.0266829)
Supplement: Attachment — Submitted filename: Response to Reviewers.docx [file pone.0266829.s005.docx]

*We thank reviewers for their thoughtful comments and suggestions that have helped to improve the manuscript. Our comments are below. Lines numbers correspond to the tracked changes version.*

Reviewer #1: Using whole-genome sequence data to examine the epidemiology of antimicrobial resistance in Escherichia coli from wild meso-mammals and environmental sources on swine farms, conservation areas, and the Grand River watershed in southern Ontario
Manuscript Number: PONE-D-21-40450

The study derived the whole-genome sequence data of Escherichia coli isolates with demonstrated antibiotic resistance phenotypes. The isolates belonged to a previous wildlife study and a public health surveillance program. In total, 200 isolates from water, wildlife, swine manure pit, and other environmental samples such as soil and dumpsters were used. The AMR genes corresponding to antibiotic resistance phenotypes were identified in silico. Plasmid incompatibility groups were also identified, more frequently among swine isolates. Although the study does not precisely identify the sources of AMR strains, certain conclusions were drawn on the frequency of occurrence of AMR strains and the genotypes, likely transfer of resistance or the acquisition of AMR genes by E. coli isolates associated with small mammals.

1. Comments:
The study relies heavily on statistical analysis for arriving at definite conclusions at the end. The MDR isolates belonging to sequence types of public health significance were isolated from a limited number of small mammals. The isolates were collected during 2011-13. Wouldn’t the resistance patterns or the distribution of E. coli have changed in these regions over the years? This is also obvious from the relatively low prevalence of MDR phenotypes and the distribution of plasmid-borne genes (e.g. β-lactamase genes) among the isolates.

*Thank you for the feedback. It's true that we lean heavily on our statistical analysis for interpretation, since we used an epidemiological approach to explore the frequencies, patterns and distributions of resistance genes and plasmid replicons in different sources, and location types. We agree that the resistance patterns or distribution of E. coli may have changed in these regions over the years, thus, we opted to account for year as a potential confounder in our analyses (see Figure 2). We've added a line in the discussion to convey the point you make (lines 610-613).*

2. The procedures for sample collection, treatment and isolation E. coli have already been published for these isolates. Therefore, this section can be significantly shortened (L133-161). Similarly, I guess L175-194 also refer to the procedures followed in 2011.

*Thank you for pointing this out, these sections have been shortened, and only the most important details have been retained (lines 155-211).*

3. The study used whole genome sequence data of a large number of isolates (n=200). However, the distribution of isolates from different sources vary widely, with most of the isolates belonging to raccoons and soil. This might have impacted the statistical analysis, since the distribution or the odds of finding a resistance gene can vary widely when the number of isolates is not uniform.

*It's true that unbalanced data can be problematic and cause modeling issues. In our analyses, we opted to reclassify sources into larger groups (Fig 1)* *to ensure adequate statistical power, and more balanced sampling of categories. We ended up with the distribution shown in Table 2 for our source type analyses: wildlife (n=73), swine manure pit (n=31), water (n=20), and "other environmental" (n=76). For the subset analysis on swine farms and conservation areas, the source distribution ended up being even: wildlife (n=73), soil (n=73). We report all 95% confidence intervals around our OR estimates to ensure it's clear to the reader that certain categories which are smaller have wider confidence intervals. Although the source distributions for the broad analysis are not ideal, our study represents a hypothesis-generating study, and we were opportunistic with available data from available sources, and careful to focus only on one variable at a time, either "location type" or "source type" (while accounting for potential confounding effects of sampling year). In line with the suggestions above, we have suggested larger sample sizes of certain source categories are needed in future work (lines 607-610).*

4. The rate and frequency of isolation of pathogenic E. coli (e.g. STEC) strains depend on the special selective enrichment regimes used for their isolation. Since no such protocols have been used for the isolation of human pathogenic E. coli groups in this study, the identified serogroups might represent only a proportion of such isolates from different sources.

*Thank you for pointing out this limitation. It has been added to the manuscript (lines 680-682).*

5. Further, there other sources of pathogenic E. coli strains such as the feces of other small animals, birds etc. Specific E. coli pathogroups are also found associated with swine. Therefore, E. coli isolates from the paw of raccoons may not simply represent “contaminants form external sources such as water or anthropogenic sources”.

*We have ensured that we have qualified our discussions of pathogenic E. coli strains with the word "potential" (lines 713-714; line 682) and maintain that our findings of certain strains nearly exclusively in conservation areas (area 1 in particular) are "suggestive" of anthropogenic exposures (line 708), which doesn't exclude other possible sources.*

6. L298-300: E. coli serovars (including O103) can often be associated with human, animal or avian sources. I agree with the authors that this serotype has caused human infections, but isn’t there a possibility that such strains can be associated naturally with the wild animals?

*We have revised the results and discussion to reflect the uncertainty of the pathogenicity of these serovars (lines 354-358; lines 614-615; lines 682-683).*

7. Based on the statistical analysis, the chances of finding tet(B) were higher among swine manure isolates, those of blaTEM-1 and sul2I genes were higher among isolates from water. Are there any specific reasons for this association? Are certain antibiotics employed in these swine farms?

*Good questions. Unfortunately, we didn't have access to antimicrobial use data on the swine farms, and have suggested it for future work (lines 583-585). We have provided a possible explanation for the higher prevalence of blaTEM-1 and sul2 genes in water (lines 555-556).*

8. In L491 it is said that tet(A) is not associated with any particular source overall. However, the following paragraph (L495-495) suggests that the tet(A) genes might have originated from the swine farms. Similarly, since tet(B) gene is more prevalent in water isolates, water was presumed to be the source of this gene in all other environments. These are contradictory. When this gene is uniformly found across different environments, how is its source and mode of dissemination predicted? Studies have shown tet genes in bacteria from pristine areas that were never exposed to antibiotics.

*Thank you for catching this error in over-interpretation of our analyses (we will need multivariable models to assess location type and source type in the same model). We inferred that primary sources of certain genes/plasmids would have a higher prevalence (this is an inference however). Uniform distributions don't tell us a whole lot, other than widespread dissemination, and that no particular source should necessarily be suspected as a primary source. We tried to simultaneously combine the broad scale analysis with the location type analysis, but this is over-interpretation, and beyond the ability of our hypothesis-generating study. We have removed the contradictory section, and revised the interpretation, and added another reference (lines 577-587).*

9. In L589-591, it is stated that small animals are at higher risk of acquiring AMR E. coil through anthropogenic contamination. L80-81 suggest small mammals could be a potential source of AMR to humans and domestic animals.

*Although these statements are not mutually exclusive, we have added several phrases to capture both concepts throughout (line 70; line 682; lines 712-713).*

10. Lines 532-533: “Resistant E. coli were significantly more likely to be detected on the paws of raccoons in conservation areas compared to swine farms”. Why is it so?

*The previous authors hypothesized that this was related to foraging behaviour by raccoons in dumpsters. We have added a line to convey this hypothesis (lines 638-639).*

11. The manuscript emphasizes on pathogenic and resistant E. coli strains being found on the paws of raccoons. However, there is always a possibility of such strains being associated with the gastrointestinal tracts of healthy raccoons. A recent study [ Orden et al., 2020. Raccoons (Procyon lotor) in the Madrid region of Spain are carriers of antimicrobial-resistant Escherichia coli and enteropathogenic E. coli. Zoonoses Public Health. 2021; 68: 69– 78] have reported the presence of pathogenic and AMR E. coli in the feces of small mammals like raccoons and suggest these as the sources of such strains to humans. Certain serovars (e.g. O77, O25) were found both on raccoon paws and faeces in your study too.

*Thank you for pointing out this reference, it was missed. We have added these findings to the discussion (lines 682-685), and highlighted the zoonotic concern of potentially pathogenic E. coli strains isolated from raccoons (lines 712-713).*

Reviewer #2: The work presented in this article contributes to a comprehensive examination of the role of wildlife in the maintenance and dissemination of AMR determinants within the Grand River watershed. The work is particularly important to identify the circulation of antimicrobial resistance genes and the mobile genetic elements that facilitate their transmission among different sources including humans. Authors could successfully relate the association of some of the antimicrobial resistance determinants to a particular environment or location types. However, the authors have not discussed or analyzed the association of phenotypic resistance and the occurrence of corresponding drug resistance genes among different sources.

*We opted to perform only a preliminary scan of class-level sensitivity and specificity of in silico identification of AMR for our hypothesis-generating epidemiologic study. The aim of this work was focused on the epidemiology of those genes in different sources and location types, thus, we argue that a fine-scaled assessment of in silico methods was not necessary for our objective. The WGS data from the wildlife study have been deposited and the phenotypic data for those isolates was previously made available by Bondo et al. (see data URLs for those previous manuscripts), should readers want to assess gene level sensitivity and specificity for these isolates. There were many possible avenues for data analysis, and we decided to focus on areas that were supported by our expertise (i.e., epidemiology). In the interest of keeping our manuscript as streamlined as possible, and minimize the addition of further tables and analyses, we opted to keep our AMR phenotype-genotype assessment as it currently is, as a secondary objective to provide a course-grained validation of our statistical analyses.*

Comments

1. Authors have used a significant amount of data from their previous study, this causes repetition of data.

*While it's true that we have used the sequenced isolates from swine farms in this manuscript, we argue these manuscripts each have a different scope and different objectives regarding the epidemiology of AMR. The previous manuscript was focused on the epidemiology of AMR in E. coli (and in Salmonella) at the swine farm level, and the analyses addressed the following predictor variables: sampling year, site id, and source type. This manuscript builds on those previous analyses and examines location type and another source (water) from a broader geographic region. The previous manuscript is focused on a small-scale, local analysis, whereas the present manuscript is focused on broader scale analyses and epidemiological patterns which may be related to different types of environments (farm vs. conservation area), and sources (i.e., water). Combining this present manuscript with our previous analyses looking at both Salmonella and E. coli would have negatively affected interpretability, presentation of the data, discussion flow, and manuscript length. The end of the introduction has been re-worked to ensure it is clear why the present work was performed, and how it differs from the previous studies (lines 97-110).*

2. Number of samples used from each source is not clearly mentioned in the methodology

*The number of isolates available for sequencing or that were previously sequenced have been added to the methods section (lines 158-161; lines 210-211).*
