# Supplementary material for: Using whole-genome sequence data to examine the epidemiology of antimicrobial resistance in Escherichia coli from wild meso-mammals and environmental sources on swine farms, conservation areas, and the Grand River watershed in southern Ontario, Canada
Source: PLoS One. 2022 Apr 8;17(4):e0266829. doi: 10.1371/journal.pone.0266829 (PMC8993012; doi:10.1371/journal.pone.0266829)
Supplement: Attachment — Submitted filename: Responses to reviewers.docx [file pone.0266829.s006.docx]

*Thank you again for your helpful feedback and insights. The lines listed here correspond to the tracked changes version.*

Reviewer #1: PONE-D-21-40450R1

The manuscript is adequately revised. A few minor points need attention.

In the abstract, it is necessary to indicate that the soil isolates were 73 and dumpster isolates were 3 considering the huge difference in the number of isolates from these two sources.

*We agree it is best to report these categories separately. The change has been made.*

Line 77: Please delete “in” after “documented”.

*Thank you for catching this error.*

Line 78: Please insert a dot after [4, 17, 18]. And start with “However,”

*The change has been made.*

An additional objective was to assess the validity of in silico identification of AMR genes, using phenotypic susceptibility test results as the gold standard. Does it mean the validity of the tool used? Does the specificity depend on the tool, targe genome or the sources?

*Interesting point. A line has been added to the discussion to elaborate on this (lines 607-611). The test performance depends largely on the AMR identification tool used, but other factors should also be considered (unlikely related to source, unless source impacts the quality of data).*

In your study, the test sensitivity for beta lactams was 90.2% and aminoglycosides, 81.8%. Is this the same as the positive predictive value?.

*The test sensitivity is a measure of the ability of the test to detect true positives (true positives that test positive/all true positives), whereas the positive predictive value is a measure of the probability of a test positive result being truly positive (true positives that test positive/all test positives). While positive predictive values have been used in this context (they are typically used in clinical medicine), I would prefer to avoid them since they are heavily influenced by the overall prevalence of the “disease” or AMR gene, in this case.*

Lines 161-181: The section under “Previous culture and susceptibility testing” . As I could understand, this data is from the previous study. In that case, the section can be merged with the previous section and reduced to few lines indicating only the antimicrobials tested. The methods, reference standards etc are available from the previous publication.

*This section has been shortened (lines 167-176), but important methodological information has been retained to ensure the reader can access the relevant information for the additional objective (validity of in silico AMR identification) within the same manuscript, without having to check the previous papers.*

Table 1: Please indicate bla genes in proper format. TEM is written as a subscript of bla and in normal font (TEM is not italicized).

*Thank you for pointing this out. The formatting has been corrected.*
